# A Natural Product from *Polygonum cuspidatum* Sieb. Et Zucc. Promotes Tat-Dependent HIV Latency Reversal through Triggering P-TEFb’s Release from 7SK snRNP

**DOI:** 10.1371/journal.pone.0142739

**Published:** 2015-11-16

**Authors:** Cong Wang, Shuiyuan Yang, Huasong Lu, Hongchao You, Man Ni, Wenjun Shan, Ting Lin, Xiang Gao, Haifeng Chen, Qiang Zhou, Yuhua Xue

**Affiliations:** 1 School of Pharmaceutical Sciences, Xiamen University, Xiamen, Fujian, China; 2 Department of Molecular and Cell Biology, University of California, Berkeley, Berkeley, CA, United States of America; Temple University School of Medicine, UNITED STATES

## Abstract

The latent reservoirs of HIV represent a major impediment to eradication of HIV/AIDS. To overcome this problem, agents that can activate latent HIV proviruses have been actively sought after, as they can potentially be used in combination with the highly active antiretroviral therapy (HAART) to eliminate the latent reservoirs. Although several chemical compounds have been shown to activate latency, they are of limited use due to high toxicity and poor clinical outcomes. In an attempt to identify natural products as effective latency activators from traditional Chinese medicinal herbs that have long been widely used in human population, we have isolated procyanidin C-13,3',3"-tri-O-gallate (named as REJ-C1G3) from *Polygonum cuspidatum* Sieb. et Zucc., that can activate HIV in latently infected Jurkat T cells. REJ-C1G3 preferentially stimulates HIV transcription in a process that depends on the viral encoded Tat protein and acts synergistically with prostratin (an activator of the NF-κB pathway) or JQ1 (an inhibitor of Brd4) to activate HIV latency. Our mechanistic analyses further show that REJ-C1G3 accomplishes these tasks by inducing the release of P-TEFb, a host cofactor essential for Tat-activation of HIV transcription, from the cellular P-TEFb reservoir 7SK snRNP.

## Introduction

The highly active antiretroviral therapy (HAART) has successfully controlled the development and progression of HIV/AIDS. However, the treatment must be maintained for life, which can lead to serious chronic consequences and extraordinary financial constraints on the overburdened health care system [1–3]. Moreover, the interruption of HAART can cause the activation of latent reservoirs of HIV, which are transcriptionally silent but remain replication-competent despite extended HAART [4]. Clearly, the HAART-mediated viral suppression alone cannot eradicate HIV and novel approaches must be designed to eliminate the latent reservoirs.

In response to this challenge, one strategy nicknamed “shock and kill” has been proposed to achieve HIV eradication in two steps [5, 6]. The “shock” phase is designed to reactivate latent proviruses, which is then followed by the “kill” phase to prevent the spread of the activated viruses by HAART and also eliminate the HIV-producing cells by immune responses and viral cytopathogenicity. The key to the successful implementation of the “shock and kill” strategy is to find specific and effective drugs for activating latent HIV. In this regard, several chemical compounds such as HMBA [7], prostratin [8] and SAHA [9] have been shown to reactivate latent HIV through different mechanisms. However, further analyses indicate that they all display strong toxicity and poor clinical outcomes [7–9]. Thus, new and improved latency activators are urgently needed at this moment.

A major rate-limiting step during HIV-1 gene expression is the elongation of RNA polymerase (Pol) II to produce full-length viral transcripts. To overcome this restriction, HIV-1 encodes an essential regulatory protein called Tat to bind to and recruit the host positive transcription elongation factor b (P-TEFb) to the 5’ end of the nascent viral RNA that folds into a stem-loop structure called the TAR element [10, 11]. Once recruited to this position, P-TEFb can phosphorylate the Pol II CTD and negative elongation factors to stimulate Pol II elongation [10, 12]. P-TEFb, consisting of CDK9 and cyclin T1 (or the minor form T2 that is not recognized by Tat), is normally sequestered in an inactive state in the 7SK snRNP [13–15]. Within this multi-subunit complex, the non-coding 7SK snRNA functions as a scaffold and the HEXIM1/2 protein acts as an inhibitor of CDK9 [16]. The sequestration of P-TEFb in 7SK snRNP has been proposed as a key factor contributing to HIV latency [5].

Although P-TEFb is necessary for Tat-transactivation, it is not sufficient for maximal Tat activity, which is required for efficient exit of HIV from latency [17]. Through sequential affinity-purification, another multi-subunit P-TEFb-containing complex termed the Super Elongation Complex (SEC) has recently been identified as the native form of human cofactor for Tat and is required for full Tat-dependent HIV transcription [18–20]. In addition to P-TEFb, the SEC also contains the well-characterized elongation stimulatory factor ELL1 or ELL2 that can synergize with P-TEFb to suppress the pausing of Pol II and stimulate the production of full-length HIV transcripts [18, 20, 21].

All the current man-made chemical latency-reversing agents have been shown to have unacceptable side effects and /or largely ineffective in early stage of clinical trials. It is in this context that we have decided to take a different route by selecting natural products derived from traditional Chinese medicinal herbs that may display latency-reversing activity. These herbs have been used widely in human population for hundreds or perhaps even thousands of years. Toward this goal, we report here the identification of a monomeric compound called REJ-C1G3 from the roots of *Polygonum cuspidatum* Sieb. et Zucc. that can efficiently reactivate HIV-1 transcription in latently infected Jurkat T cells. *Polygonum cuspidatum* Sieb. et Zucc. has traditionally been used for the treatment of jaundice, inflammation, hyperlipidemia, and certain infectious diseases [22–24]. REJ-C1G3 isolated from extracts of this medicinal herb was found to preferentially promote the Tat-dependent HIV-1 transcription and synergize with prostratin and JQ1, two conventional chemical latency activators, to reactivate latent HIV. Our mechanistic studies indicate that it may accomplish these functions by causing the release of P-TEFb from 7SK snRNP, the cellular reservoir of excess and unused P-TEFb.

## Results

### Extracts of *Polygonum cuspidatum* Sieb. et Zucc. contain an activity that promotes HIV-1 LTR-driven EGFP expression

**Fig 1:**
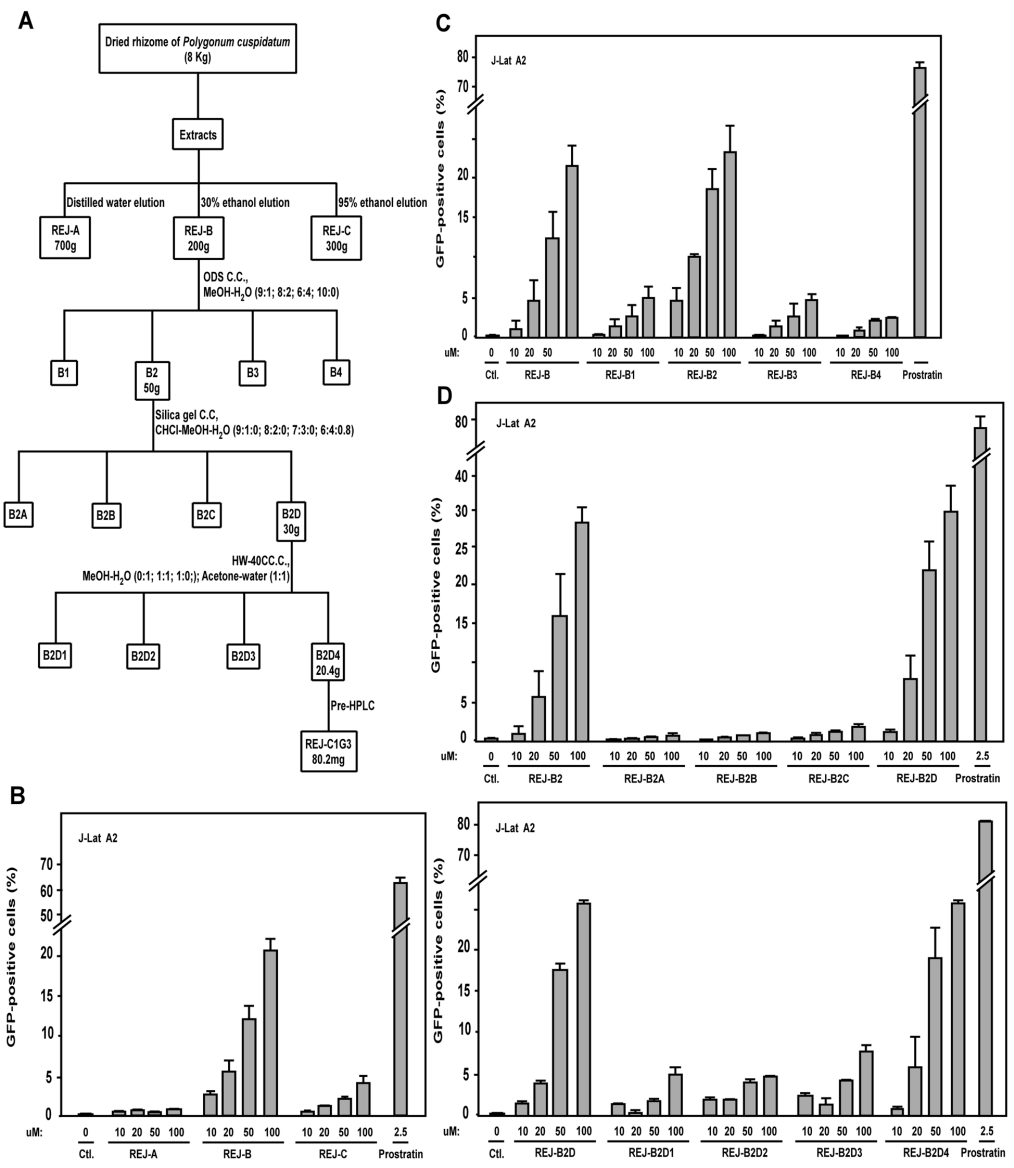
Extracts of *Polygonum cuspidatum* Sieb. et Zucc. contain an activity that promotes HIV-1 LTR-driven EGFP expression. **(A)** A flow chart depicting the purification of the active product REJ-C1G3 from the extracts of *Polygonum cuspidatum* Sieb. et Zucc.. **(B-E)** During the purification of REJ-C1G3, individual fractions obtained after each chromotography step were tested for their abilities to activate EGFP expression in J-Lat A2 cells. The indicated concentrations of these fractions or 2.5 μM prostratin as a positive control were incubated with J-Lat A2 cells for 12 hr, and the EGFP-positive cells detected by flow cytometry. The percentages of EGFP-expressing cells in the entire viable cell population were shown, with the error bars in all panels indicating mean ± SD from three independent experiments.

To identify natural products derived from traditional Chinese medicinal herbs that can cause HIV latency reactivation, we used the Jurkat T cell line-based J-Lat A2 cells as a model system. This cell line contains an integrated 5’-LTR- Tat-Flag-iRES-EGFP-3’-LTR expression cassette that is normally silent but produces the enhanced green fluorescent (EGFP) protein upon activation [25]. To screen for active natural products, J-Lat A2 cells were first incubated individually with thousands of partially purified fractions derived from a collection of over 100 traditional Chinese medicinal herbs that are included in a library in the School of Pharmaceutical Sciences at Xiamen University. The activation of the LTR-driven EGFP production was detected by flow cytometry. During the screen, the 30% ethanol elution (called REJ-B) from a HP-20 macroporous absorbent resin column that was loaded with the extracts from the dried rhizome of *Polygonum cuspidatum* Sieb. et Zucc. (Fig 1A) was found to dose-dependently activate EGFP expression in J-Lat A2 cells (Fig 1B).

In order to identify the exact component of REJ-B that was responsible for HIV latency activation, this fraction was subjected to further purification according to the scheme depicted in Fig 1A (the detailed procedure is described in Materials and Methods). After each purification step, the eluted fractions were evaluated for their abilities to activate EGFP expression in J-Lat A2 cells, and Fig 1C–1E show a representation of such evaluations. Using this strategy, an active product called REJ-C1G3 was isolated.

### REJ-C1G3 is identified as Procyanidin C-13,3',3"-tri-O-gallate

**Fig 2:**
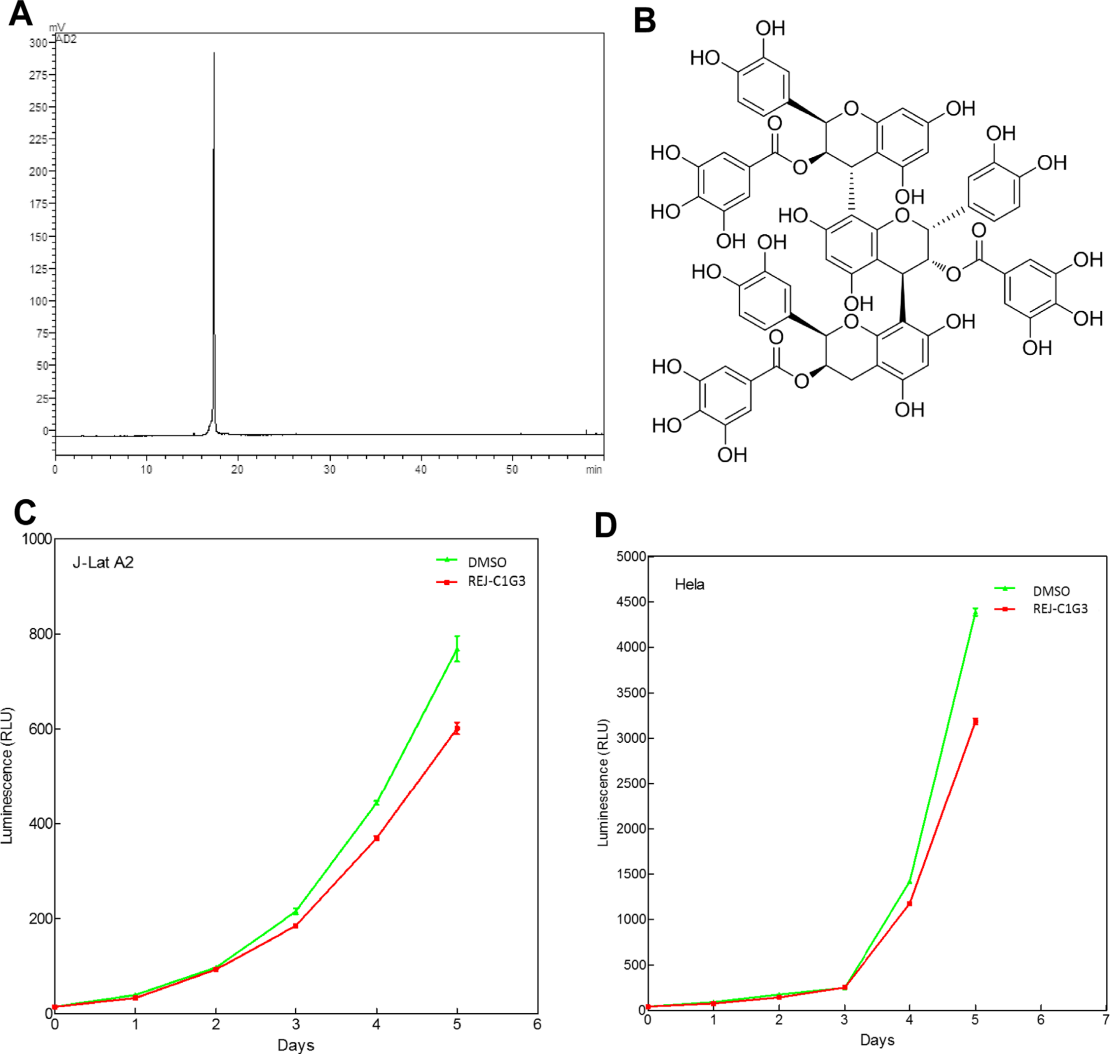
Determination of the purity and structure of REJ-C1G3 and its impact on cell viability. **(A)** High performance liquid chromatography (HPLC) was performed to confirm the purity of REJ-C1G3. **(B)** Chemical tructure of REJ-C1G3. **(C and D)** J-Lat A2 (C) and HeLa cells (D) were exposed to REJ-C1G3 (20 μM) for 5 days and then assayed for cell viability. The error bars represent mean ± SD from three independent experiments.

Obtained as a tan amorphous powder, REJ-C1G3 was subsequently analyzed by HPLC for its purity (Fig 2A). More structural information was obtained from further analysis of the mass spectral fragmentation pathways of REJ-C1G3 (S1 Fig). These results were consist with the analysis of similar structure from literature [26]. 1H-NMR (ACETONE-D6 + D2O, 600MHz) data were also consistent with the literature data [27] (the detailed procedure is described in Materials and Methods) and the structure of REJ-C1G3 is thus determined as Procyanidin C-13,3',3"-tri-O-gallate (Fig 2B).

To determine whether REJ-C1G3 may affect cell growth, we used the Promega CellTiter-Glo Luminescent Cell Viability Assay kit to measure the viability of both J-Lat A2 and HeLa cells that were treated with REJ-C1G3 for up to 5 days. As indicated in Fig 2C and 2D, the treatment had very minimal effect during the initial 4 days. Since the REJ-C1G3 treatment never exceeded 24 hours in all the experiments performed in the current study, we concluded that the effect of REJ-C1G3 on proviral activation is not due to any significant change to cell growth and viability that may result from long-term exposure to this product.

### REJ-C1G3 activates HIV-1 transcription in a dose- and time-dependent manner

**Fig 3:**
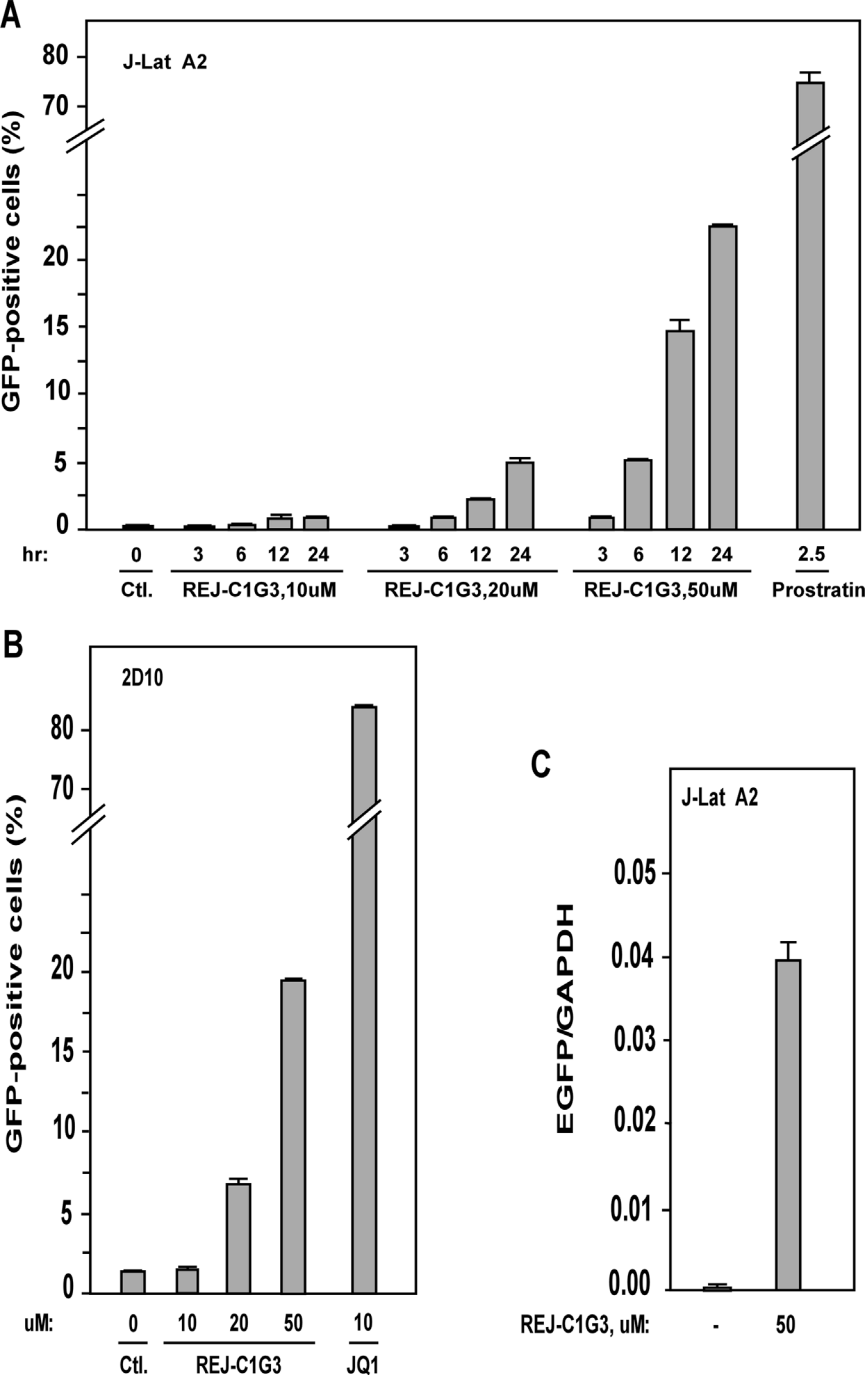
REJ-C1G3 activates HIV-1 transcription in a dose- and time-dependent manner. **(A)** J-Lat A2 cells were incubated with increasing concentrations (0, 10, 20, and 50 μM) of REJ-C1G3 and for increasing time periods (3, 6, 12, and 24 hr) as indicated. Prostratin (2.5 μM) was used as a positive control. The treated cells were analyzed by flow cytometry for EGFP-producing cells, with the error bars representing mean ± SD from three independent measurements. **(B)** 2D10 cells were treated with the indicated concentrations of REJ-C1G3 or JQ1 which was used as a positive control. After 24 hr, the cells were analyzed by flow cytometry for EGFP-producing cells. The error bars indicate mean ± SD from three independent experiments. **(C)** The EGFP and GAPDH mRNA levels in treated J-Lat A2 cells were determined qRT-PCR and the EGFP/GAPDH ratios are shown. The error bars represent mean ± SD from three independent experiments.

To test the activity of REJ-C1G3, we treated J-Lat A2 cells with increasing concentrations of this product and also for different time periods. The percentage of EGFP-positive cells increased in a dose- and time-dependent manner (Fig 3A). Representative FACS analyses used to generate Fig 3A are shown in supplementary data (S2 Fig). Importantly, the same stimulatory effect of REJ-C1G3 was also found in the Jurkat-based post-integrative latency model 2D10 [28] (Fig 3B), which is another well-characterized latency model harboring almost the complete HIV genome with only the *nef* gene replaced by that encoding EGFP.

To confirm that the enhanced EGFP signals detected by FACS were due to the elevated expression of EGFP mRNA, quantitative RT-PCR (qRT-PCR) with primers that hybridize to a distal portion of the *EGFP* gene was performed. As indicated in Fig 3C, REJ-C1G3 indeed reactivated the HIV provirus through stimulating viral mRNA production.

### REJ-C1G3 predominantly stimulates Tat-dependent HIV-1 transcription

**Fig 4:**
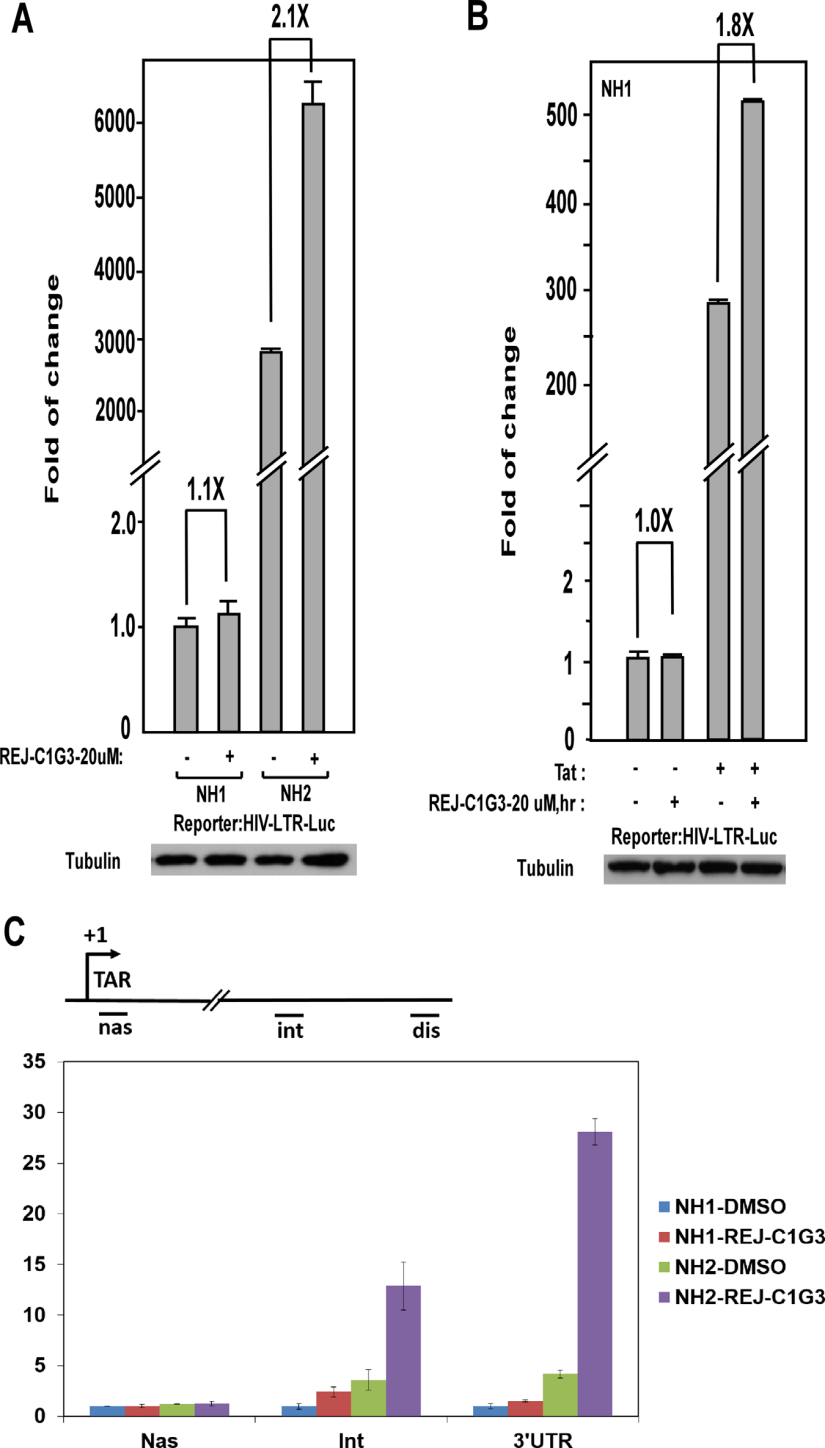
REJ-C1G3 preferentially stimulates the Tat-dependent HIV transcription. **(A)** The HeLa-based based NH1 and NH2 cells were treated with REJ-C1G3 or DMSO as indicated. WCE were prepared and tested for luciferase activities (top panel) and the celluar tubulin levels detected by Western blotting (bottom panel). The activity detected in cells treated with DMSO was set to 1. The error bars represent mean ± SD from three independent experiments. **(B)** NH1 cells were transciently transfected with an empty vector (-) or the Tat-expressing construct (+; 10 ng/well) and then treated with REJ-C1G3 or DMSO (-) as indicated. WCE were prepared and tested for luciferase activities as in A. (C) mRNAs transcribed from the integrated HIV-1 LTR-luciferase reporter gene were analyzed by qRT-PCR with primers that hybridize to the three marked locations along the DNA template and then normalized to the levels of GAPDH mRNA. The error bars represent the mean ± SD from three independent measurements.

To make sure that the REJ-C1G3-induced HIV-1 transactivation was dependent on the viral 5’-LTR but not any other unrelated viral or non-viral sequences in the integrated expression cassette, we tested the effect of REJ-C1G3 on expression of an integrated luciferase reporter gene that is driven solely by the HIV-1 5’-LTR in the HeLa-based NH1 cell line [29] To our surprise, REJ-C1G3 (20 μM for 12h) had little effect on the LTR-driven luciferase expression in NH1 cells (1.1-fold), but when a construct expressing the HIV-1 Tat protein was stably transfected into these cells to generate NH2 cells [29], REJ-C1G3 was found to activate the LTR more potently in this isogenic cell line (2.1-fold; Fig 4A). The same observation was also obtained by transiently transfecting the Flag-Tat expression vector into NH1 cells (Fig 4B). Together, these results indicate that the stimulatory effect of REJ-C1G3 is exerted through the HIV-1 5’ LTR and that the product preferentially stimulated the Tat-dependent HIV-1 transcription but not basal, Tat-independent transcription.

To determine whether the effect of REJ-C1G3 on HIV transcription was at the stage of initiation or elongation, a quantitative RT-PCR (qRT-PCR)–based assay was performed to measure the relative abundance of HIV mRNAs at several different locations downstream of the viral transcription start site. As shown in Fig 4C, REJ-C1G3 had little effect on the production of very short, just initiated transcript, but produced the greatest effect on generating the long transcripts especially in the Tat-containing NH2 cells. Thus, REJ-C1G3 stimulated the Tat-dependent HIV-1 transcription at the elongation but not initiation phase.

### REJ-C1G3 synergistically reactivate latent HIV-1 with prostratin or JQ1

The data presented so far have revealed that the activation of HIV-1 transcription by REJ-C1G3 depended on the viral Tat protein. In addition to the dependence on the Tat/TAR/P-TEFb-SEC pathway that is stimulated by REJ-C1G3, NF-κB is another important transcription factor that also contributes significantly to the activation of HIV-1 LTR and latency [8]. Prostratin, a frequently studied latency activator, acts as a PKC agonist to enhance the NF-κB pathway to induce HIV transactivation [8].

**Fig 5:**
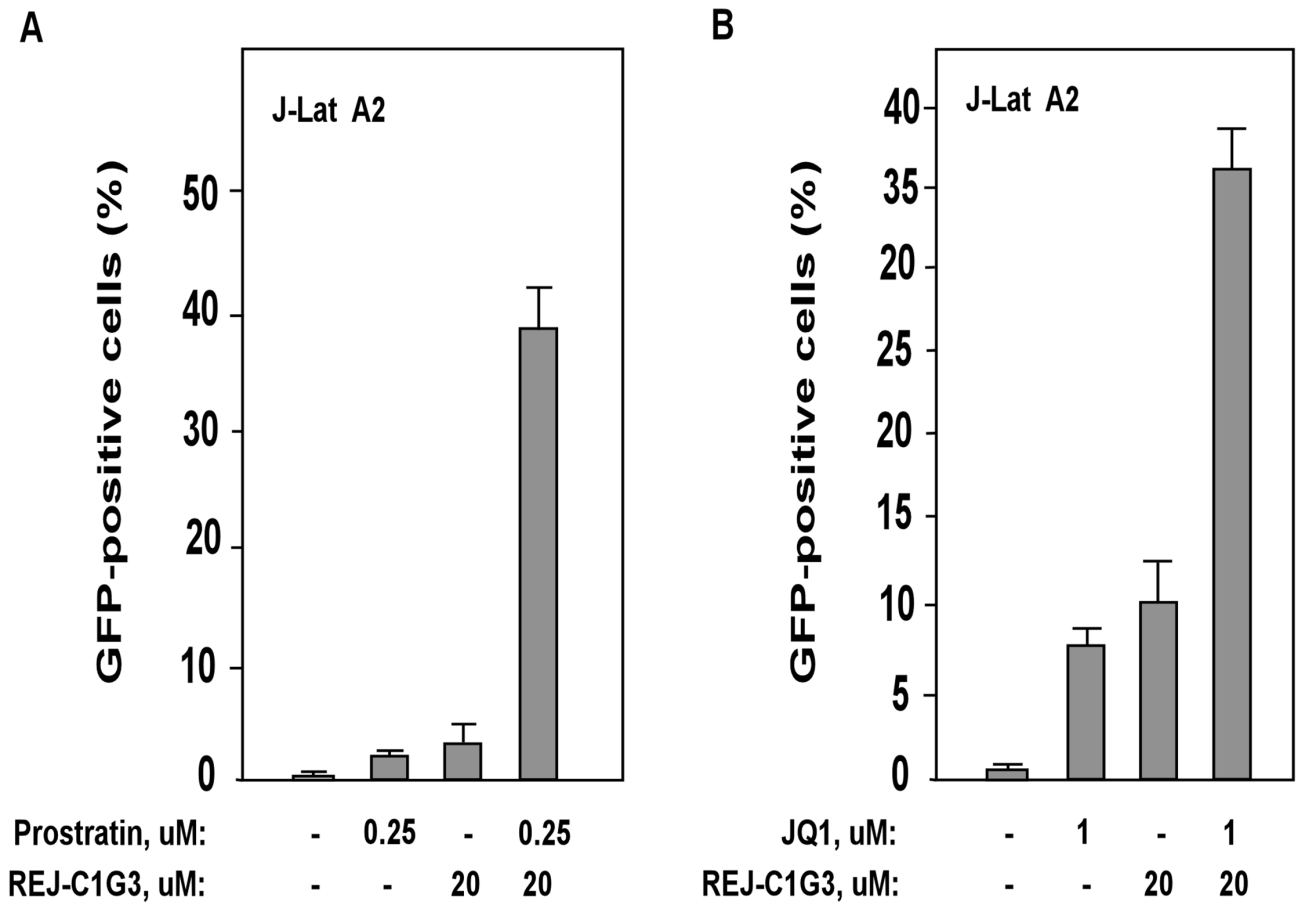
REJ-C1G3 synergizes with prostratin or JQ1 to reactivate latent HIV. **(A and B)** J-Lat A2 cells were treated with REJ-C1G3 (20 μM) alone or in combination with prostratin (0.25 μM) or JQ1 (1 μM) for 12 hr and then analyzed by flow cytometry for the percentages of EGFP-positive cells in the total cell population. DMSO was used as a negative control. The error bars represent mean ± SD from three independent experiments.

Likely owing to their abilities to target different signaling pathways, prostratin and REJ-C1G3 were found to synergistically activate HIV-1 transcription in J-Lat A2 cells (Fig 5A). When used separately, 0.25 μM prostratin and 20 μM REJ-C1G3 induced only 2.7% and 4.2% EGFP-positive cells, respectively. However, the combination of the two produced 39.0% EGFP-positive cells, demonstrating that the two activators produced a much greater activation level than the sum of the effects produced by either activator alone (Fig 5A). In addition to prostratin, we also observed a strong synergism between REJ-C1G3 and JQ1 (Fig 5B). JQ1 is a BET bromodomain inhibitor that acts by antagonizing the inhibitory effect of Brd4 on Tat-transactivation and recently proposed as a novel latency-reversing agent [29, 30]. Thus, the combinatorial use of REJ-C1G3 together with the conventional chemical latency activators such as prostratin and JQ1 may be an effective way to reverse HIV latency with reduced toxicity to cells.

### REJ-C1G3 promotes release of P-TEFb from 7SK snRNP

**Fig 6:**
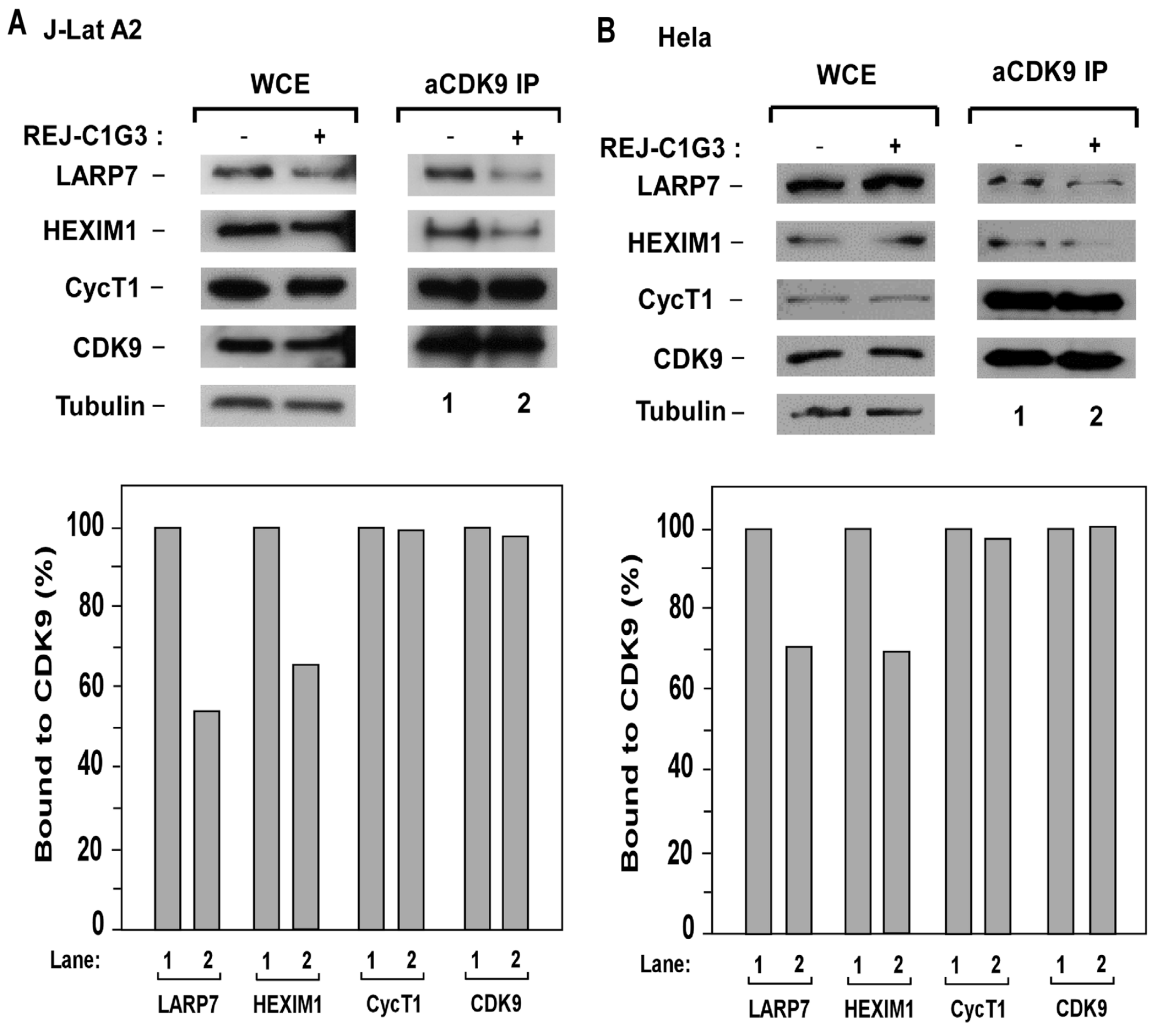
REJ-C1G3 triggers the release of P-TEFb from 7SK snRNP. **(A and B)** WCE of J-Lat A2 (A) HeLa cells (B) that were either untreated (-) or treated (+) with REJ-C1G3 (20 μM) were analyzed by Western blotting for the indicated proteins (left panels). Anti-CDK9 immunoprecipitates (IP) derived from WCE were tested by Western blotting for presence of the indicated proteins (right panels). The amounts of the CDK9-bound proteins were quantified by densitometry and shown at the bottom. CDK9 in untreated cells (lane 1) was artifically set at 100%.

P-TEFb plays a key role in HIV-1 transcription and its sequestration in the inactive 7SK snRNP has been proposed to contribute to viral latency [5]. To determine whether REJ-C1G3 may affect the level of P-TEFb present in 7SK snRNP, we performed anti-CDK9 immunoprecipitation in J-Lat A2 cells and HeLa cells that were treated with REJ-C1G3 and examined the association of CDK9 with two signature 7SK snRNP components HEXIM1 and LARP7 in the immunoprecipitates. As shown in Fig 6A and 6B, reduced amounts of HEXIM1 and LARP7 but not CycT1 were found to bind to CDK9 upon the treatment with REJ-C1G3. These results indicated that the treatment caused the disassociation of P-TEFb from the 7SK snRNP.

## Discussion

Up to this point, HAART has been recognized as the most effective way to treat HIV infection. However, the HAART-mediated viral suppression alone cannot eradicate the latent HIV reservoirs and novel approaches must be designed to accomplish this difficult goal in order to cure infected patients of HIV/AIDS [31]. The popular “Shock and Kill” strategy designed to eradicate latent HIV relies on the discovery of effective agents that can reactivate the latent reservoirs without significantly impacting the host cell growth and function [5, 6, 32]. Unfortunately, the currently available latency activators have all shown high toxicity and/or poor clinical outcomes [7–9].

To search for effective latency activators, many European and North American companies and laboratories are screening various chemical libraries composed of mostly synthetic small molecule compounds. The work described here complements this conventional approach by focusing on natural products derived from traditional Chinese medicinal herbs, many of which have seen centuries of usage and are well tolerated in a wide spectrum of human population. It is in this context that REJ-C1G3 from *Polygonum cuspidatum* Sieb. et Zucc. has been identified and shown to activate HIV latency by preferentially enhancing Tat-dependent HIV transcription. Although REJ-C1G3 may not necessarily be the eventual drug used clinically for waking up latent proviruses and curing HIV/AIDS, its identification and characterization in the present study serve as an important proof of concept that traditional Chinese medicinal herbs can be a good source for finding such drugs.

The data presented in the current study indicate that the REJ-C1G3-induced latency activation is likely caused by the release of P-TEFb from 7SK snRNP and slightly enhanced formation of the SECs that are key for Tat-transactivation. Since Tat and P-TEFb/SECs are well known to stimulate the elongation stage of Pol II transcription [18], it is not surprising to see that REJ-C1G3, which promotes HIV elongation, strongly synergized with prostratin [33], which acts on the PKC pathway to stimulate the NF-κB-mediated viral transcription initiation [29], in inducing latency activation.

It is not clear at this moment how REJ-C1G3 triggers the release of P-TEFb from 7SK snRNP and it is likely this natural product activates a certain cellular signaling pathway that eventually leads to the release. So far, several signaling pathways that trigger specific changes in the modification patterns of the 7SK snRNP components have been proposed to cause the disruption of 7SK snRNP and release of P-TEFb [11, 34, 35]. Future studies are necessary to determine whether REJ-C1G3 may regulate any of these pathways to alter the balance of P-TEFb distributed in between the inactive 7SK snRNP and the transcriptionally active SECs.

## Materials and Methods

### General TLC

Precoated silica gel GF254 plates (SiO2; Qingdao Haiyang Chemical Industry). Column chromatography (CC): (SiO2, 60–100 and 200–300 mesh, Qingdao Haiyang Chemical Industry), Sephadex LH-20 gel (Pharmacia Biotek), and ODS (40–63μm; YMC, Japan). HPLC: Shimadzu Prominence LC-20A liquid chromatography, with LC-20AT pumps, SPD-20A UV detector (Shimadzu Co., Japan) as well as YMC-Pack ODS-A column (250mm×10mm, 5μm) (YMC Co., Japan). NMR Spectra: BrukerAvance 600 III spectrometer;δ in ppm, with Me4Si as internal standard; J in Hz. ESI-MS:Q Exactive mass spectrometer(Thermo Scientific).

### Plant Material

The rhizome of *Polygonum cuspidatum* Sieb. et Zucc. were collected from Fujian province, P. R. China, and were authenticated by Engineer Xiu-Hong Zou, Forestry Bureau of Yongchun, P. R. China.

### Data of the compound

The fragment resulting from the loss of a gallic acid (-C7H4O4) from negative molecular ion [M-H] was deduced by the ions at m/z 1169.2 as well as the loss of a fragment of phloroglucinol heterocyclic fragment (-C6H6O3) from ion m/z 1169.2 led to the MS peak at m/z 1043.1. The fragment ions at m/z 1169.2 could also be broken to lose an epicatechin, a gallic acyl fragment (-C7H4O4), agallic acid group fragment (-C7H6O5) to give ions at m/z 881.5, m/z 729.1 and m/z 711.1. The ion at m/z 729.1 may further lose a gallate group fragment (-C7H6O5) to obtain ion at m/z 557.1. The fragment ions at m/z 1169.2 could still be broken to lose a gallate group fragment (-C7H6O5) to give ion at m/z 999.2, which may further lose a gallic acid fragment (-C7H4O4) to give ion at m/z 847.1.

δ: 7.04,6.99,6.93 (each2H, s, galloy-H), 6.4–6.9 (9H, m, B ring-H), 5.9–6.18 (4H, m, A-ring-H), 5.69 (3H, m, 2,3’, 3”- H), 5.52 (1H, brs, 3-H), 5.28 (1H, brs, 2’-H), 5.19 (1H, brs, 2”-H), 4.99 (2H, brs, 4 and 4’-H), 2.79–2.91 (2H, m, 4”-H)

### Extraction and isolation of REJ-C1G3

Dried rhizome of *Polygonum cuspidatum* Sieb. et Zucc. (8kg) was boiled and refluxed with 60% ethanol for 2 h (30 L × 3 times). After filtration, the extracted solution was evaporated under reduced pressure and the condensate was subjected to HP20 macroporous resin column chromatography and eluted with water, 30% ethanol, 95% ethanol to afford REJ-A, REJ-B and REJ-C. REJ-B was applied to ODS column chromatography and eluted with MeOH-H2O elution (1:9, 1:4, 2:3 and 1:0) to give four fractions (REJ-B1 ~ REJ-B4). REJ-B2 was chromatographed by silica gel column using stepwise gradient elution with CHCl3-MeOH-H2O (9:1:0.1, 8:2:0.2, 7:3:0.5 and 6:4:0.8) to obtain four subfractions (REJ-B2A ~ REJ-B2D). REJ-B2D was further separated by resin gel HW-40C column chromatography eluting with methanol and aqueous acetone (50%) and the50% aqueous acetone elution was further purified through preparative HPLC eluting with 82% aqueous acetonitrile to afford compound 1 (80.2mg)

### Antibodies

The antibodies against CDK9, HEXIM1, LARP7, CycT1 have been described previously [18, 34].

### Co-immunoprecipitation assay

The co-immunoprecipitation assay was performed essentially as described [18] with minor modifications. Whole cell extracts (WCE) were prepared with a buffer containing 20mM HEPES-KOH [Ph7.9], 5mM EDTA, 0.5% NP-40, 3mM dithiothreitol, 0.5mM PMSF and 0.3M NaCl. WCE were then incubated with 2μg of the indicated antibodies overnight, which was followed by incubation with protein-A beads (Life Invitrogen) for 3 hr. The beads were washed extensively with buffer D0.3M (20mM HEPES-KOH [Ph7.9], 15% glycerol, 0.2mM EDTA, 0.2% NP-40, 1mM dithiothreitol, 1mM PMSF and 0.3M KCl), eluted with 0.1M glycine [pH 2.0], and analyzed by Western blotting with the indicated antibodies.

### Flow cytometry-based screening

J-Lat A2 cells were incubated with the various fractions generated during the purification of the active product from *Polygonum cuspidatum* Sieb. et Zucc.. The concentrations and time periods of the treatment are indicated in the figures or figure legends. After the incubation, cells were harvested, washed and resuspended in phosphate-buffered saline (PBS). Flow cytometry was performed in both J-Lat A2 cells and 2D10 cells as previously described [25]

### Luciferase assay

For the luciferase assay, the HeLa-based NH1 and NH2 cell lines [29] containing an integrated HIV-1 LTR-luciferase reporter gene without (NH1) or with (NH2) the Tat-expressing plasmid were used. Luciferase activities were measured using kit E1501 from Promega and following the manufacturer’s instructions. Lysates were prepared from approximately equal number of cells and normalized based on their contained α-tubulin levels.

### Cell viability

Measurement of cell viability was performed with the Promega CellTiter-Glo kit according to the manufacturer’s instructions. Cells were seeded at 3000 cells/well in a 96-well plate (3 wells/sample) and then treated or untreated with REJ-C1G3. The measurements were taken at several time points to track cell proliferation during the course of the treatment.

### Quantitative PCR

The reactions were performed with Applied Biosystems Real-Time PCR System and CWBIO UltraSYBR Mixture RT-PCR reagents following the manufacturers’ instructions. The sequences of the primers used in PCR are: EGFP-F: CAGTGCTTCAGCCGCTACCC; EGFP-R: AGTTCACCTTGATGCCGTTCTT; GAPDH-F: GCACCACCAACTGCTTAGC; GAPDH-R: GGCATGGACTGTGGTCATG; Nascent RNA-F: GTTAGACCAGATCTGAGCCT; Nascent RNA-R: GTGGGTTCCCTAGTTAGCCA; Interior-F: CTCTTTCGAAAGAAGTCGGGG; Interior-R: GAACAACTTTACCGACCGCG; 3’ UTR-F: GCTATTAATAACTATGCTCAAAAAT; 3’ UTR-R: CAACAACAATTGCATTCATTTTATG. PCR conditions included an initial denaturing step at 95°C for 10 min and then 40 cycles of amplification. Each cycle consisted of 15 sec at 95°C and 1 min at 62°C. The values were normalized to those of GAPDH to obtain the relative folds of induction.

### Ethics statement

No specific permissions were required for the described study, which fully complies with all the relevant laws and regulations. The rhizome of *Polygonum cuspidatum* Sieb. et Zucc. is a traditional Chinese medicinal herb, which is widely cultivated in China. This study does not involve any endangered or protected species.

## Supporting Information

S1 Fig MS analysis of REJ-C1G3. High performance liquid chromatography-mass spectrometry (HPLC-MS) was conducted to help determine the molecular formula of compound 1.
(TIF)

S2 Fig REJ-C1G3 reverses HIV latency in J-Lat A2 cells (related to Fig 3A). Representative FACS plots that were used to generate Fig 3A. Analysis was gated on live cells according to forward and side scatter. EGFP-expressing cells were compared to the DMSO-treated cells, and the percentages of EGFP-expressing cells in the whole cell population were shown.
(TIF)

S3 Fig REJ-C1G3 synergizes with JQ1 to reactivate latent HIV provirus. Representative FACS plots that were used to make Fig 3B. J-lat A2 cells were treated with REJ-C1G3 (20 μM) together with JQ1 (1 μM) or DMSO as a negative control for 12 hr and then analyzed by flow cytometry for the percentages of EGFP-positive cells in the whole viable cell population.
(TIF)
