# Supplementary figures and images for: A Natural Product from Polygonum cuspidatum Sieb. Et Zucc. Promotes Tat-Dependent HIV Latency Reversal through Triggering P-TEFb’s Release from 7SK snRNP
Source: PLoS One. 2015 Nov 16;10(11):e0142739. doi: 10.1371/journal.pone.0142739 (PMC4646521; doi:10.1371/journal.pone.0142739)

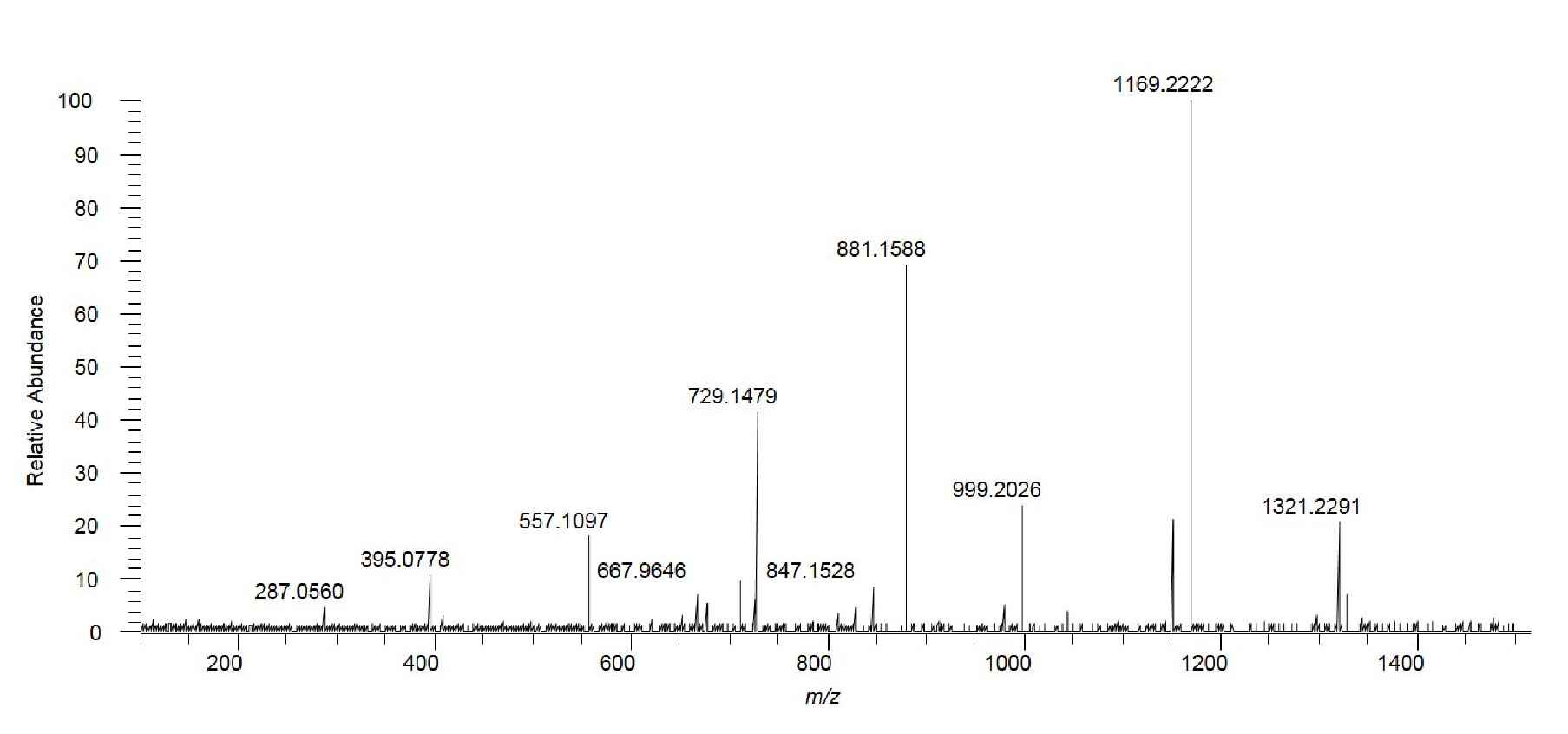

Supplement: S1 Fig — High performance liquid chromatography-mass spectrometry (HPLC-MS) was conducted to help determine the molecular formula of compound 1. (TIF) [file pone.0142739.s001.tif]

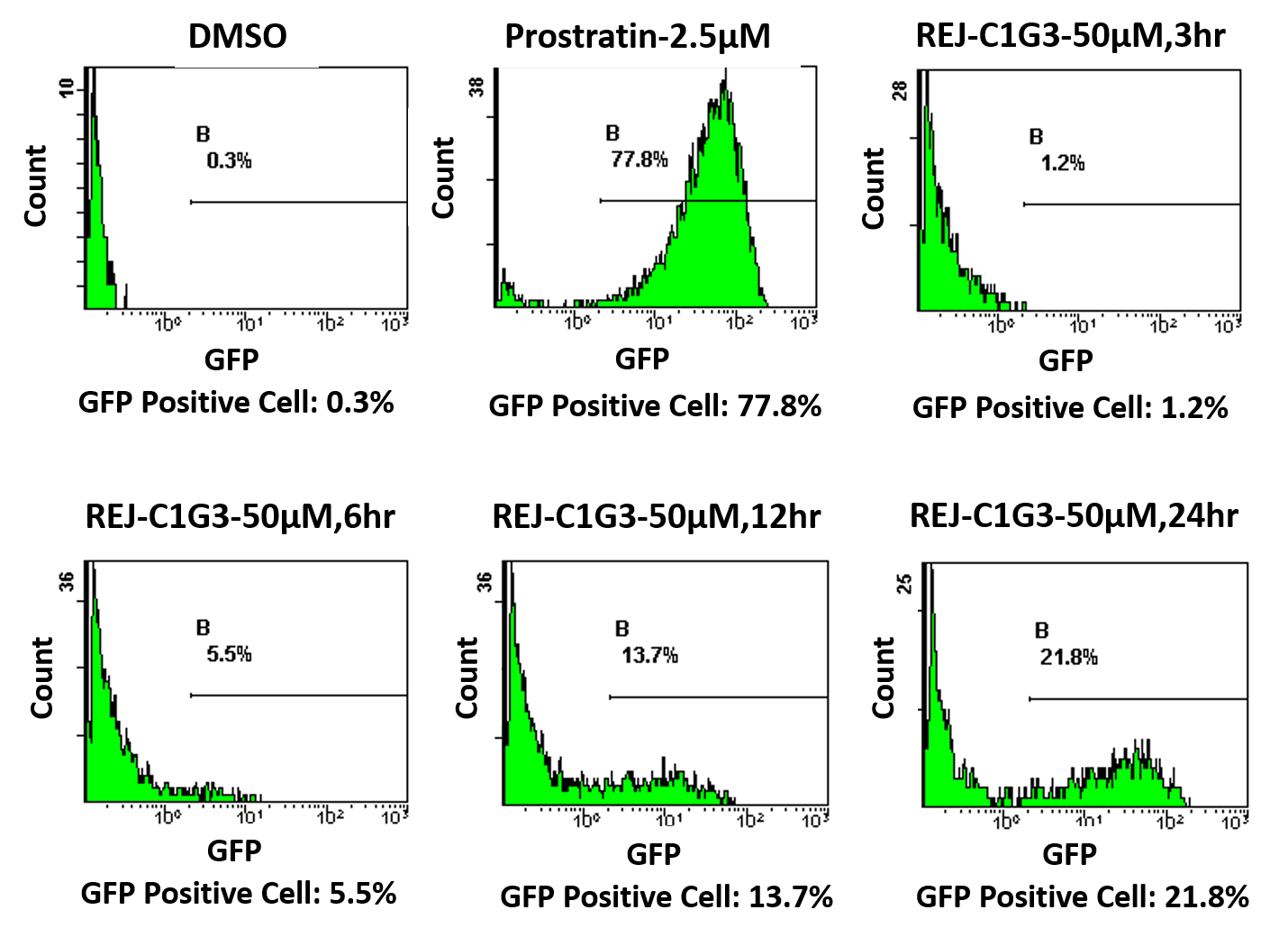

Supplement: S2 Fig — Representative FACS plots that were used to generate Fig 3A. Analysis was gated on live cells according to forward and side scatter. EGFP-expressing cells were compared to the DMSO-treated cells, and the percentages of EGFP-expressing cells in the whole cell population were shown. (TIF) [file pone.0142739.s002.tif]

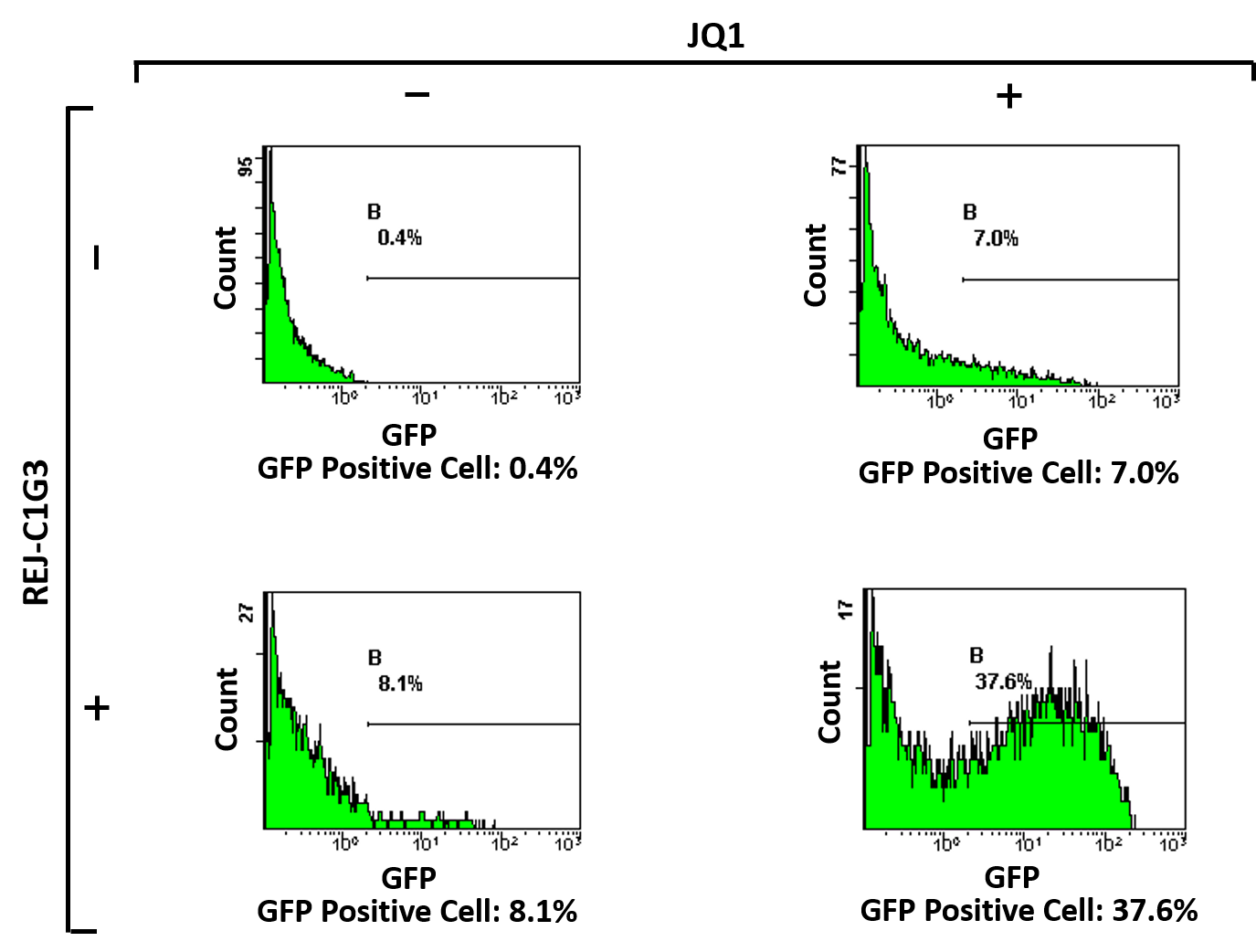

Supplement: S3 Fig — Representative FACS plots that were used to make Fig 3B. J-lat A2 cells were treated with REJ-C1G3 (20 μM) together with JQ1 (1 μM) or DMSO as a negative control for 12 hr and then analyzed by flow cytometry for the percentages of EGFP-positive cells in the whole viable cell population. (TIF) [file pone.0142739.s003.tif]
